# Heavier Lies Her Crown: Gendered Patterns of Leader Emotional Labor and Their Downstream Effects

**DOI:** 10.3389/fpsyg.2022.849566

**Published:** 2022-08-29

**Authors:** Andrea C. Vial, Colleen M. Cowgill

**Affiliations:** Department of Psychology, Division of Science, New York University Abu Dhabi, Abu Dhabi, United Arab Emirates

**Keywords:** emotional labor, gender, power, leadership, emotion, prosocial behavior

## Abstract

Women use power in more prosocial ways than men and they also engage in more emotional labor (i.e., self-regulate their emotions to respond and attend to the needs and emotions of other people in a way that advances organizational goals). However, these two constructs have not been previously connected. We propose that gendered emotional labor practices and pressures result in gender differences in the prosocial use of power. We integrate the literature on emotional labor with research on the psychology of power to articulate three routes through which this happens. First, women may be more adept than men at the intrapersonal and interpersonal processes entailed in emotional labor practices—a skill that they can apply at all hierarchical levels. Second, given women’s stronger internal motivation to perform emotional labor, they construe power in a more interdependent manner than men, which promotes a more prosocial use of power. As a result, female powerholders tend to behave in more prosocial ways. Third, when they have power, women encounter stronger external motivation to engage in emotional labor, which effectively constrains powerful women’s behaviors in a way that fosters a more prosocial use of power. We discuss how, by promoting prosocial behavior among powerholders, emotional labor can be beneficial for subordinates and organizations (e.g., increase employee well-being and organizational trust), while simultaneously creating costs for individual powerholders, which may reduce women’s likelihood of actually attaining and retaining power by (a) making high-power roles less appealing, (b) guiding women toward less prestigious and (c) more precarious leadership roles, (d) draining powerful women’s time and resources without equitable rewards, and (e) making it difficult for women to legitimize their power in the eyes of subordinates (especially men). Thus, emotional labor practices can help explain the underrepresentation of women in top leadership positions.

## Introduction

When they have power, women tend to behave in more prosocial ways than men. For example, a meta-analysis of 162 studies by Eagly and Johnson (1990) found a stronger tendency in women than in men to lead in an interpersonally oriented style in laboratory experiments. Across studies, women in positions of authority were more likely than men in those positions to prioritize the maintenance of interpersonal relationships, to tend to the morale and welfare of others, or to show consideration for others—e.g., helping and doing favors for subordinates. Subsequent meta-analyses confirmed these early conclusions (van Engen and Willemsen, 2004). Clearly, many female powerholders show no shortage of care for others, even when some scholars have argued that power can unleash self-serving and often destructive behavior that is insensitive to the needs of other people (e.g., Anderson et al., 2003; Keltner et al., 2003; Van Kleef et al., 2008; Lammers et al., 2012; Rucker and Galinsky, 2016). But what makes female powerholders more interpersonally sensitive than their male counterparts? And what are the consequences of women’s tendency to wield power “with a velvet glove” (i.e., in a more prosocial way)?

We posit that the answer to these questions partly lies on gendered patterns of *emotional labor*—which we define as the act of self-regulating one’s emotions to respond and attend to *others’* needs and emotions in a manner that advances organizational goals (Hochschild, 1983; Grandey, 2000; Cheung and Tang, 2010). We argue that women in power behave in a more prosocial manner than men because they have a stronger tendency to practice emotional labor. These gender^1^ differences have a mixed set of consequences: On the one hand, women’s more prosocial use of power can be beneficial for subordinates and organizations. On the other hand, we argue that gendered emotional labor practices can simultaneously create costs for individual powerholders—especially when emotional labor is externally motivated—and undermine gender equality in top leadership positions in multiple ways. Here, we integrate the previously disconnected literatures on emotional labor and the psychology of power to articulate three routes through which this happens. We contribute to existing models of gender and leadership by identifying emotional labor as a key construct that can illuminate why men and women express power differently and why it is more difficult for women to attain and retain powerful roles (Vial et al., 2016).

Power is often defined as the extent to which an individual exerts asymmetric control or influence *over others* (Schmid Mast et al., 2009)—for example, having the authority to issue orders that others must follow, or controlling access to valued resources (Magee and Frasier, 2014)—while also being free *from others*, or having the discretion to operate autonomously, unfettered by the will and needs of other people (Fast et al., 2009; Inesi et al., 2011; Lammers et al., 2016). Ostensibly, then, power runs counter to emotional labor, as the latter prioritizes accommodating the emotions of other people rather than imposing one’s own views independently from others (Rucker and Galinsky, 2016). Conversely, we propose that when people in power engage in emotional labor, this practice may foster a more prosocial use of power, one that is considerate of others and that promotes or protects their welfare (Batson, 2012). We contribute to the literature on the psychology of power (e.g., Sassenberg et al., 2014; Sturm and Antonakis, 2015; Tost and Johnson, 2019; Foulk et al., 2020) by identifying emotional labor practices as an important antecedent to prosocial power use—one that can help explain why men and women in high-power roles may behave differently.

**FIGURE 1:**
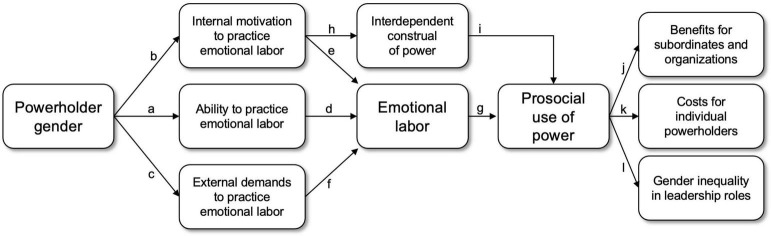
Theoretical model connecting gendered patterns of emotional labor with the prosocial use of power and its downstream consequences. Powerholder gender is related to differences in the ability (*path a*) and the internal motivation (*path b*) to practice emotional labor, as well as in external emotional labor demands (*path c*). These three factors directly contribute to emotional labor (*paths d–f*), which underlies the prosocial use of power (*path g*). Additionally, a stronger internal motivation to practice emotional labor is associated with a more interdependent construal of power (*path h*), which influences the tendency to enact power in prosocial ways (*path i*). The prosocial use of power has benefits for subordinates and organizations (*path j*), but it can also create costs for individual powerholders (*path k*) and undermine gender equality in leadership roles (*path l*).

We argue that gender differences in emotional labor practices and demands translate into women’s more prosocial use of power in at least three ways (Figure 1). First, women have a *stronger ability* than men to practice emotional labor (Figure 1, *path a*), a skill that underlies the prosocial use of power. Second, women have a *stronger internal motivation* than men to perform emotional labor (Figure 1, *path b*), which may lead women to construe power in an interdependent manner that fosters prosocial rather than self-serving behavior. And third, women face *stronger external demands* than men to practice emotional labor (Figure 1, *path c*), which constrain powerful women’s ability to exercise their authority in self-serving ways. In the sections that follow, we integrate the literatures on emotional labor and the psychology of power to develop a theoretical framework in which we articulate these three pathways connecting emotional labor to female powerholders’ prosocial use of power (Figure 1, *path g*) as well as positive and negative consequences for individuals, groups, and gender equality at large (Figure 1, *paths j-l*). We begin by discussing gender differences in emotional labor and then review research that supports the claim that women are more likely than men to behave in a prosocial way when they occupy high-power roles.

## What Is Emotional Labor and What Are Its Antecedents?

A variety of social settings have tacit (and sometimes explicit) norms or “display rules” for what is an acceptable or desirable emotional expression. These norms delineate socially appropriate ways of interacting with others, including whether and to what degree felt emotions should be expressed (Ekman and Friesen, 1975; Matsumoto, 1990). At its core, emotional labor is the regulation of felt and expressed emotions (whether effortful or not) to match these emotional display rules with the objective of fulfilling organizational goals (Hochschild, 1983; Grandey, 2000; Cheung and Tang, 2010). People practice emotion regulation in a wide variety of contexts (e.g., students at school, spouses at home; Gross, 1998); however, *emotional labor* specifically occurs when people deploy emotion regulation strategies to meet organizational goals (Grandey, 2000). For example, when an employee in a service job continues to smile to an angry customer to prevent them from leaving the establishment, or when an employee masks their feelings of frustration during a long staff meeting to preserve harmony within the team. Indeed, emotional labor has tangible organizational benefits, as it fosters an atmosphere in which people feel at ease, valued, and understood (Iszatt-White, 2009).

Emotional labor is enacted on two different levels. One of these levels is strictly *intrapersonal* and involves self-regulation and expression of the right kind and amount of emotion (Totterdell and Holman, 2003; Hülsheger and Schewe, 2011). To achieve this, individuals resort to one of two strategies: (a) a response-focused emotion regulation strategy known as “surface acting,” which entails the suppression of felt negative emotions through the modification of facial displays (e.g., “putting on a smiley face”); and (b) an antecedent-focused emotion regulation strategy known as “deep acting” that involves changing inner feelings through cognitive reappraisal—for example focusing on positive rather than negative feelings (Hochschild, 1983; Grandey, 2000, 2003; Cheung and Tang, 2010). In addition to these intrapersonal processes, emotional labor is enacted on an *interpersonal* level. In order to respond adequately to the emotions of another person, one must first identify how that person is feeling (Ashtar et al., 2021). Thus, although an important part of the process is self-oriented, emotional labor is inherently other-oriented as it requires one to be attuned to others’ emotions and to accommodate and cater to those emotions (i.e., paying attention to, accurately recognizing, and responding effectively to the feelings of others). Interpersonal and intrapersonal processes can take place sequentially and repeat several times in the course of a single interaction (e.g., identify another person’s feelings; self-regulate one’s own emotions; produce the appropriate response; identify the person’s reaction; recalibrate or maintain one’s emotional expression, and so on).

When individuals practice emotional labor (e.g., by being attentive to the emotional experience of others and by self-regulating to respond to those emotions in a way that facilitates organizational goals), such practices can manifest in an interdependent and relational approach and in a wide variety of behaviors. These behaviors, which essentially grease the wheels of human interaction in organizational settings, can be classified into three broad categories, including (a) acting in prosocial rather than self-serving ways (e.g., being interpersonally helpful; showing concern for others’ welfare at work; guiding, comforting, and defending others); (b) being sensitive to others’ views (e.g., seeking out their opinion; allowing them to voice opposing perspectives; accommodating others’ needs); and (c) maintaining a positive emotional environment (e.g., making others feel at ease and valued; tending to their morale; providing them with emotional support).^2^

### Antecedents of Emotional Labor: Ability and Motivation

When considering the psychological antecedents of emotional labor, it is important to distinguish between the ability to practice it (Figure 1, *path a*) and the motivation to engage in it (Figure 1, *path b* and *path c*). One may be skilled at practicing emotional labor but not be particularly motivated to do so—either generally or in a specific context. Alternatively, one may desire to engage in emotional labor and fail miserably. This distinction is key to understanding how emotional labor practices relate to gender, as research suggests that women may be more skilled at behaviors relevant to emotional labor than men as well as more strongly motivated to practice them. Similarly, it is crucial to distinguish between emotional labor that springs from internal motivation (Figure 1, *path b*) and emotional labor that is externally motivated (Figure 1, *path c*): Whether one genuinely wishes to cater to others’ emotions (for instance, out of an *empathic concern* for others; Batson, 1987, 2011), or whether one feels an external demand to do so (e.g., due to formal work-role requirements; Hochschild, 1983; Grandey, 2000; Cheung and Tang, 2010).

These distinctions between ability, internal, and external motivation form the basis of three different routes in our theoretical model through which gender differences in emotional labor result in women’s stronger prosocial use of power. According to social role theory (Wood and Eagly, 2002; Eagly and Wood, 2012), gender differences and similarities in social behavior are the product of the disparate distribution of women and men into distinct social roles. For instance, women occupy the great majority of care-oriented roles in society. Such gendered distribution of labor, which is due in part to evolved physical differences between women and men (e.g., in terms of size, strength, reproductive activities), influences their behavior through various processes, including the creation of gender roles. These gender roles dictate different expectations for women and men—for example, the kinds of psychological characteristics they are believed to possess. Given that women tend to occupy care-oriented roles, they are expected to possess the psychological characteristics that are best suited to those roles, such as being highly communal, kind, cooperative, etc. These expectations, as well as the requirements of the specific roles that women and men occupy, shape their social behavior (see Eagly et al., 2000) via hormonal processes, socialization practices (i.e., how women and men are taught to behave from a young age), and social regulation (e.g., penalties and rewards for behaving in ways that contradict or uphold expectations, respectively). As we discuss below, gender differences have been identified in the ability, internal motivation, and external demands to practice emotional labor, which reflect the influence of gendered social roles (Wood and Eagly, 2002; Eagly and Wood, 2012).

#### Gender Differences in Ability to Practice Emotional Labor

Some evidence suggests that women may be more adept than men at the intrapersonal processes entailed in emotional labor practices, as they usually engage in a wider range of emotion regulation strategies than men (Garnefski et al., 2004). For instance, fMRI studies indicate that they use positive emotions in the service of reappraising negative emotions to a greater degree (McRae et al., 2008). With regards to the more interpersonal aspects of emotional labor, studies indicate that women possess better *empathic accuracy* than men—the ability to correctly infer what another person is thinking or feeling (Ickes et al., 2000). Women are also more successful than men at deciphering others’ non-verbal cues (La France et al., 2009; Williams et al., 2009), and at recalling people’s non-verbal cues and facial expressions (Hall et al., 2007). Other research has revealed that women score higher than men on interpersonal aspects of emotional intelligence, which involves the ability to perceive and express emotion and regulate emotion in the self and others (Mayer et al., 1999), including social skills such as being perceptive, empathic, and adaptable (Argyle, 1994; Petrides and Furnham, 2000; Joseph and Newman, 2010; Cabello et al., 2016).

In sum, empirical evidence indicates that women may be more skilled than men at a host of behaviors that constitute the building blocks of emotional labor. In line with social role theory (Wood and Eagly, 2002; Eagly and Wood, 2012), these ability differences may reflect women’s historical tendency to occupy positions in which emotional skills are paramount (e.g., care-oriented roles). As care-oriented roles promote and require emotional abilities, such abilities may become entrenched into the female gender role in a way that shapes women’s behavior (Eagly et al., 2000). For instance, the socialization of young girls may place a stronger emphasis than that of boys on the development of emotional skills such as being able to identify and name emotions (McClure, 2000; Brody and Hall, 2010).

However, as alluded to earlier, an ability to practice emotional labor successfully should not be confused with a motivation to do so. This distinction becomes particularly important when considering emotional labor practices that are externally motivated (as discussed below), because ability and motivation in this case may have opposite effects on well-being. Specifically, whereas being more adept at managing emotions could make emotional labor feel effortless, a strong external pressure to do so may take a psychological toll, reducing emotional autonomy and feelings of authenticity (Brotheridge and Grandey, 2002). We discuss these burdens in detail in the section on *The Downstream Consequences of Women’s Prosocial Use of Power*.

#### Gender Differences in Internal Motivation to Practice Emotional Labor

Both internal (i.e., intrinsic) and external (i.e., extrinsic) motivation to practice emotional labor to achieve organizational goals may be stronger in women than in men. With regards to internal motivation, women may genuinely have a stronger proclivity than men to both consider and accommodate the emotions of others. For example, women appear to care more than men about correctly reading and responding to others’ emotions (Ickes et al., 2000; Klein and Hodges, 2001). Women are also generally more likely than men to engage in the kinds of non-verbal behaviors that facilitate social interaction (for a review, see LaFrance and Vial, 2016), such as smiling (LaFrance et al., 2003), maintaining eye contact (Hall, 1984); keeping closer physical proximity (Hall and Gunnery, 2013); orienting their body more directly toward their interaction partners (Helweg-Larsen et al., 2004); employing *affiliative speech*, or language that affirms and shows support to the other person (Leaper and Ayres, 2007); and using back-channel responses (e.g., head nodding) to signal that they are listening (Leaper and Robnett, 2011). Other research suggests that women (but not men) may generally internalize prosocial rather than self-interested behavior as their intuitive response to other people (Rand et al., 2016).

As discussed in the previous sections when describing the basic tenets of social role theory (Wood and Eagly, 2002; Eagly and Wood, 2012), although the origins of these gender differences are likely multiply determined, one clear source can be found in different socialization practices that, from a young age, encourage girls more so than boys to cultivate communal attributes (Brody, 1993; Hibbard and Buhrmester, 1998; Shields, 2002; Chaplin et al., 2005). Women consistently report a stronger communal self-concept than men, viewing themselves as friendlier, less selfish, and more concerned with others (e.g., Witt and Wood, 2010; Hsu et al., 2021). Thus, the evidence indicates that women are more highly internally motivated than men to practice emotional labor.

#### Gender Differences in External Emotional Labor Demands

In addition to having stronger internal motivation, women may also experience stronger external pressures than men to get emotional labor right. Whereas some individuals may be more adept at emotional labor than others and/or personally more inclined to practice it, organizational norms often impinge on people’s ability to express their emotions freely. Indeed, one strong antecedent to emotional labor is the extent to which there are contextual pressures that create a sense of obligation to practice it, or *emotional labor demands*. Organizational contexts and roles vary in how much emotional labor they require. Women are generally more likely than men to hold jobs entailing high work-role demands to display positive emotions either to customers and clients or to coworkers and superiors (Guy and Newman, 2004; Cortes and Pan, 2018). In many female-dominated (i.e., “pink collar”) occupations, workers are expected to employ emotional skills to bring about organizational ends, whereas workers in male-dominated occupations do not face these demands (Meier et al., 2006; Johnson and Spector, 2007; Nixon, 2009). Indeed, the concept of emotional labor was originally developed by Hochschild (1983) to describe the experience of low-level service jobs (e.g., flight attendants, customer-oriented roles), which do not offer much opportunity for advancement up the organizational hierarchy, and which continue to be occupied primarily by women (U.S. Bureau of Labor Statistics, 2021).

Importantly, not only are women more likely than men to work in occupations with strong emotional labor demands; they are also more likely than men to encounter pressures to practice emotional labor even when occupying the same organizational roles (e.g., Schaubroeck and Jones, 2000). From the perspective of social role theory (Wood and Eagly, 2002; Eagly and Wood, 2012), cultural gender stereotypes develop from observation of the historical distribution of women and men into different social roles, leading people to *expect and require* women to accommodate others’ needs and emotions to a greater extent than men. Indeed, there is a strong belief that women, more so than men, tend to care about and be sensitive to the feelings of other people (Prentice and Carranza, 2002; White and Gardner, 2009; Haines et al., 2016). Stereotypes around emotion expression in particular portray women as well-suited to comply with emotional display rules that require gauging and responding to others’ emotions (Lopez-Zafra and Gartzia, 2014) and expressing positivity and interpersonal sensitivity (Shields, 2002; Timmers et al., 2003; Fischbach et al., 2015). People tend to implicitly associate the expression of anger with men (Bijlstra et al., 2010; Neel et al., 2012; Smith et al., 2015); indeed, men are commonly stereotyped as aggressive or violent (Leach et al., 2017) and as more likely than women to display negative emotions such as anger and hostility (Plant et al., 2000; Shields, 2000)—behaviors that are incompatible with emotional labor practices.

Gender stereotypes have a strong prescriptive component (Burgess and Borgida, 1999; Prentice and Carranza, 2002). Thus, people typically think that women *should* be caring, kind, and careful with others’ emotions. They do not require the same of men, who are held to a lower communality standard in general (Biernat and Manis, 1994; see also Vial and Cimpian, 2020, for a review of shifting gender standards and social rewards). Unsurprisingly, then, women are judged in relation to a higher standard than men with respect to performing emotional labor at work. For example, an experiment showed that women do not reap any special rewards for being interpersonally helpful with coworkers, whereas men receive accolades for the same behavior (Heilman and Chen, 2005; see also Farrell and Finkelstein, 2007). Women are expected to show positive emotions in general more than men (Hess et al., 2005) and their emotional expression at work is scrutinized more closely (Smith et al., 2016). In contrast, men’s emotional expression is judged based on a relaxed standard: Whereas women elicit penalties from other people when they express anger in a professional context and their anger is viewed as unjustified, men’s anger in the same context is seen as acceptable and warranted (Brescoll and Uhlmann, 2008; Barrett and Bliss-Moreau, 2009; see also Raymondie and Steiner, 2021). In sum, even in the same organizational role, women encounter stronger external pressures than men to practice emotional labor, and are punished when they do not heed them—even when they occupy high-power roles (as we elaborate on the section on *The Pressure Route: Emotional Labor Demands Curb Women’s Self-Interested Use of Power).*

## Emotional Labor at the Top of the Hierarchy: Women’s Prosocial Use of Power

Individuals practice emotional labor at all levels of the organizational hierarchy. Whereas, in its origins, the concept of emotional labor was focused on workers at lower hierarchical levels (Hochschild, 1983), those at the top of the hierarchy also practice emotional labor—identifying others’ emotions and self-regulating their own in order to produce the kind of response that may best achieve their organizational goals. Indeed, emotional labor can be an important part of leadership (e.g., Gardner et al., 2009; Humphrey, 2012). This may be particularly the case for management roles embedded in occupational contexts that have a strong care orientation (e.g., healthcare, early education) in which communal attributes and the capacity to nurture others are viewed as more typical in leaders (Yoder, 2001; Cowgill and Vial, 2022). Unsurprisingly, women are better represented in leadership positions in these organizational contexts compared to non-care-oriented occupations (U.S. Bureau of Labor Statistics, 2021).

### Emotional Labor at the Top Translates Into Prosocial Use of Power

Given the unique behavioral affordances of high-level roles (e.g., the prerogative to impose authority over others; Magee and Frasier, 2014), we argue that emotional labor in these roles translates into a more prosocial use of power. By “prosocial use of power,” we mean a broad range of actions intended to benefit people in the organizational context other than the powerholder (i.e., behaviors such as helping, comforting, sharing, cooperation, etc.; Batson, 2012), which are supported by emotional labor practices that allow for the accurate detection and accommodation of others’ emotional needs. Prosocial attitudes and behaviors are generally valued as key features of effective leaders (e.g., Gerzema and D’Antonio, 2013, 2017; Gartzia and van Knippenberg, 2016). Indeed, promoting cooperative relationships with and among followers is often highlighted as an important leader function (De Cremer and van Knippenberg, 2002).

At the top of the hierarchy, emotional labor practices may translate into a leadership style that is more interpersonally oriented, one that draws less on dominance to influence others and, as a result, increases positive interpersonal behaviors among subordinates (Humphrey, 2012; Kakkar and Sivanathan, 2021). Emotional labor practices among managers and supervisors manifest in prosocial behaviors toward employees (e.g., helping and doing favors for subordinates), showing sensitivity to their views (e.g., not dominating a team interaction; listening and taking subordinates’ concerns into account when making decisions), and seeking to foster a positive, friendly work environment that is psychologically safe (e.g., avoid expressing anger or being too critical or too dominant; showing empathy; promoting cooperative relationships with and among followers). At the same time, those with decision-making power are often expected to self-regulate in order to maintain emotions at bay and keep a cool head to make decisions rationally—for example, to suppress feelings of empathy evoked by a specific individual in order to maximize aggregate outcomes for the group or organization they lead (e.g., Uhlmann et al., 2013). Thus, powerholders’ effective emotional labor practices entail walking a fine line between showing sensitivity and empathy and being accommodating, while at the same time not letting emotions cloud their judgment. These actions require those in high-power roles to carefully read others’ emotions and manage their own—often involving substantial self-regulation—in the service of effective communication and producing the right state of mind in others (i.e., emotional labor; Humphrey, 2012). All of these practices appear to be more common among high-power women compared to high-power men, as we describe next.

### Women Wield Power in More Prosocial Ways Than Men

Whether due to a stronger ability (e.g., Garnefski et al., 2004), internal motivation (e.g., Ickes et al., 2000), or external demand (e.g., Heilman and Chen, 2005), we argue that women’s greater likelihood to practice emotional labor results in a more prosocial use of power when they wield it compared to men (Figure 1, *path g*). Powerholders who engage in emotional labor practices are often described as *transformational* leaders (Wolfram and Mohr, 2010; Vinkenburg et al., 2011) or as *servant* leaders (Barbuto and Gifford, 2010; Lemoine and Blum, 2021), who enact a communally oriented leadership style in which individual consideration (“seeing” and nurturing followers) is key. Research has consistently found that women are more likely than men to adopt these kinds of interpersonally oriented leadership styles (Eagly and Johnson, 1990; Eagly and Johannesen-Schmidt, 2001). For example, an early study revealed that women showed more concern for others than men even when they occupied high-status organizational roles (Moskowitz et al., 1994). Meta-analytic evidence indicates that women in positions of authority are more likely than men in those positions to prioritize the maintenance of interpersonal relationships, to tend to the morale and welfare of others, or to show consideration for others—e.g., helping and doing favors for subordinates (van Engen and Willemsen, 2004). Other work suggests that women (but not men) in high-power roles are sensitive to other people’s views and perspectives, and less likely to dominate team interactions (Brescoll, 2011). Further supporting these trends, a study commissioned by LeanIn.org and McKinsey and Company (Thomas et al., 2021), which included responses from over 65,000 employees in 423 companies in the United States and Canada, revealed that female managers were more likely than male managers to provide emotional support to employees and to help them navigate work-life challenges. In the context of academia, surveys have found that female faculty perform significantly more uncompensated internal service than male faculty, acquiescing to participate in committee-work more often, even when controlling for rank (i.e., tenure; Guarino and Borden, 2017).

Thus, the existing evidence indicates that women behave in more prosocial ways than men when they have power. Nevertheless, it would be helpful for future investigations to examine this possibility more directly as well as the connection with emotional labor practices. Studies may test whether gender differences in emotion self-regulation among male and female powerholders predicts the latter’s stronger tendency to behave prosocially. Emotion regulation takes time and effortful control (Grandey, 2000); thus, studies could examine whether gender differences in powerholders’ prosocial behavior are eliminated in conditions that might impair emotional labor (e.g., under time constraints or cognitive load). Given that women are more skilled at emotional labor than men (Ickes et al., 2000; Garnefski et al., 2004; McRae et al., 2008; Cabello et al., 2016), future studies could also examine whether female powerholders respond better than male powerholders to emotional labor demands.

### Multiple Routes to Gender Differences in the Prosocial Use of Power

We propose that gender differences in the ability (e.g., Garnefski et al., 2004), internal motivation (e.g., Ickes et al., 2000), and external demands (e.g., Heilman and Chen, 2005) to practice emotional labor constitute three distinct pathways or routes through which gendered emotional labor practices and demands result in gender differences in the prosocial use of power. Of these three routes, the “ability” route (Figure 1, *paths a, d*, and *g*) is the most straightforward, as we describe below. We also propose that there are two other routes connecting gendered emotional labor practices with the prosocial use of power, which are less obvious but equally (or perhaps even more) influential: a “construal” route (Figure 1, *paths b, h*, and *i*) and a “pressure” route (Figure 1, *paths c, f*, and *g*).

#### The Ability Route: Women’s Greater Aptitude for Emotional Labor Facilitates the Prosocial Use of Power

The ability route focuses on gender differences in the ability to practice emotional labor (Figure 1, *path a*), as previously discussed (Ickes et al., 2000; Garnefski et al., 2004; McRae et al., 2008; Cabello et al., 2016). Such differences in ability may logically underlie gender differences in actual emotional labor (Figure 1, *path* d). Specifically, women’s greater aptitude for the skills involved in emotional labor relative to men would enable them to practice it, thereby directly influencing women’s prosocial use of power (Figure 1, *path g*). Women’s superior ability to accurately understand what others are feeling (Ickes et al., 2000) and read their non-verbal expressions (La France et al., 2009; Williams et al., 2009) would make it easier for them to subsequently accommodate their needs (e.g., to display the kind of individualized consideration that is central to transformational leadership styles; Eagly and Johnson, 1990; Eagly and Johannesen-Schmidt, 2001). Supporting this view, studies have found that the ability to perceive and respond to others’ emotions is positively related to behaving altruistically toward others (Charbonneau and Nicol, 2002). Thus, women may use power more benevolently than men simply because they are better equipped to practice emotional labor.

#### The Construal Route: Women’s Greater Internal Motivation to Practice Emotional Labor Translates Into a More Benevolent View (and Use) of Power

In addition to directly promoting prosocial behavior by increasing the amount of emotional labor practiced (Figure 1, *paths b, e* and *g*), we propose that women’s stronger internal motivation than men to perform emotional labor may foster prosocial behavior indirectly through a “construal” route: Due to their internal motivation to practice emotional labor (Figure 1, *path b*), women construe power in a more interdependent way than men (Figure 1, *path h*), which fosters a prosocial use of power (Figure 1, *path i*). Beyond the objective degree of power that a person may have (i.e., how much a person is actually in control of their own and others’ fate), how a person *construes* their power is fundamental to understanding how they wield it (Sassenberg et al., 2014; Sturm and Antonakis, 2015; Tost and Johnson, 2019; Foulk et al., 2020). Specifically, power appears to magnify preexisting individual dispositions to be more self-oriented or, conversely, more communally oriented (e.g., Côté et al., 2011; Galinsky et al., 2016). Those with power are highly attuned to features in their environment that can help them achieve their goals (Keltner et al., 2003; Guinote, 2007, 2008), including interpersonal and prosocial goals. When people feel powerful, they are better able to connect with and enact their true selves (Kraus et al., 2011; Kifer et al., 2013). Some may approach power in a more “personalized” way that highlights autonomy and dominance over others, whereas some may approach power in a more “socialized” manner that highlights the powerholder’s responsibility to ensure the best possible outcomes for the group at large (Frieze and Boneva, 2001; see also Wang and Sun, 2016).

Prosocial effects ensue when powerholders construe their power in interdependent ways that highlight responsibility for the welfare of others (Overbeck and Park, 2006; Gordon and Chen, 2013; De Wit et al., 2017). When people hold a more interdependent self-construal, they use power benevolently (Howard et al., 2007), and when they have a stronger other-orientation they tend to be fairer in their dealings with others (Blader and Chen, 2012). Powerholders with a stronger need to belong or be accepted tend to downplay their power and yield to the opinions and views of other people (Rios et al., 2015). Other work has shown that power can sometimes increase perspective-taking (Hall et al., 2005, 2007; Schmid Mast et al., 2009). The more the powerholder understands the high-power position as empathic and other-oriented, the more he or she is interpersonally sensitive (Chen et al., 2001; Schmid Mast et al., 2009; Côté et al., 2011; Chin et al., 2013). If the psychological experience of power leads powerholders to behave more in line with their other-oriented or self-oriented dispositions (e.g., Kraus et al., 2011; Foulk et al., 2020), then it is possible that baseline gender differences in the internal motivation to perform emotional labor may persist even when men and women occupy positions of power, and women’s propensity to act on such motivation may be enhanced. Indeed, recent investigations provide indirect evidence in line with the idea that women may think of people at the top of the hierarchy as being particularly adept at managing others’ emotions—that they may view emotional labor as central to power and leadership (e.g., Vecchio and Boatwright, 2002; Bellou, 2011; Hays, 2013; Collins et al., 2014; Gino et al., 2015).

First, given that power facilitates goal pursuit (Keltner et al., 2003; Guinote, 2007, 2008), women’s stronger communal goals (Diekman et al., 2011) and internal motivation to practice emotional labor may translate into a more prosocial use of power in alignment with those goals, whereas men’s more agentic goals may result in strong self-oriented behavior. Due to their strong emphasis on connection, interpersonal sensitivity, and the overall tendency to see oneself in a relational manner (Josephs et al., 1992; Cross and Madson, 1997; Witt and Wood, 2010; Hsu et al., 2021), women who acquire power may be overall more attuned to the needs and emotions of others, and willing and emboldened to cater to them. Compared to women, men in power may be more content with the possession of the means to dominate or impose their will onto others (i.e., “being feared”; Hays, 2013), as they generally have a more independent self-construal (Guimond et al., 2006) and tend to self-describe as more dominant and assertive (Prentice and Carranza, 2002; Hentschel et al., 2019). For example, in a series of studies, Gino et al. (2015) found that men were more likely than women to desire a highly dominant type of power, “to have an impact on, control or manage other people, influence other people, or control resources others depend on” (Gino et al., 2015).

Second, women appear to have stronger expectations than men for emotional labor in authority figures, which may mirror their differential approach to wielding power when they have it themselves. To illustrate, a meta-analysis of 69 studies drawing from three different research paradigms testing gender stereotypical perceptions of leaders and authority figures revealed that men tend to construe leadership as more agentic and less communal than women (Koenig et al., 2011). Women more than men tend to view arrogance or being controlling as undesirable characteristics of those in powerful roles, and instead value communal, positive emotional attributes in leaders (Vial and Napier, 2018). Other research has similarly revealed that, compared to men, women expect leaders to be more “people-oriented” (Bellou, 2011) and more relational (Boatwright and Forrest, 2000), and react more positively to leaders who behave more communally (Collins et al., 2014) and who show considerateness toward others (Vecchio and Boatwright, 2002). Thus, women more than men appear to envision the ideal powerful person as someone who is able to relate in a positive emotional manner to other people and to accommodate their feelings and interests; namely, someone who performs emotional labor. This vision may influence how women themselves wield power, leading to more prosocial behavior in female powerholders than in male powerholders.

#### The Pressure Route: Emotional Labor Demands Curb Women’s Self-Interested Use of Power

Finally, the third and last route is a “pressure” route such that, to some extent, observed gender differences in the prosocial use of power reflect subtly coercive emotional labor demands and looming social threats that impinge on women more strongly than on men (Figure 1, *path c*). Specifically, we propose that, although attaining structural power could free individuals to behave in more self-serving ways (Kipnis, 1972; Keltner et al., 2003; Van Kleef et al., 2008), the stronger emotional labor demands imposed on women compared to men (e.g., Heilman and Chen, 2005; Brescoll and Uhlmann, 2008; Barrett and Bliss-Moreau, 2009) do not cease as they accrue power. These demands may effectively constrain powerful women’s (but not men’s) ability to exercise their authority in self-serving ways, resulting in more prosocial power use.

Women in top roles are often expected to be more emotionally available and more sensitive to others than men in similar roles. For example, Schaubroeck and Jones (2000) showed that, within the same large organization, women perceived a stronger requirement to express positive emotions relative to men, even when position tenure and salary level were kept constant (see also Bellas, 1999). In another study, participants expected female leaders to be particularly more effective than male leaders at “caretaking” leader behaviors such as encouraging, assisting, praising, mentoring, and providing resources to others (Prime et al., 2009). Comparable expectations of caretaking and nurturing behaviors have been documented in the realm of academia, where female professors are subject to stronger emotional labor demands from students than male professors (e.g., to do special favors; El-Alayli et al., 2018). Women more than men are expected to adopt a communally oriented style of leadership focused on nurturing followers that involves listening, showing empathy, and providing emotional support to subordinates, commonly known as “servant leadership” (Barbuto and Gifford, 2010; Lemoine and Blum, 2021). Similarly, people expect women more than men to lead in a “transformational” way (Embry et al., 2008; Vinkenburg et al., 2011; Stempel et al., 2015), a leadership style that includes a strong component of consideration and support for subordinates’ needs, preferences, and welfare, and the creation of a friendly work environment that is psychologically safe (Rafferty and Griffin, 2004). At a basic cognitive level, research indicates that people expect feminine-faced leaders to be cooperative and display a prosocial leadership style based on altruism, empathy, and reciprocity, whereas they expect masculine-faced leaders to display a dominant style (Spisak et al., 2012).

Expectations that female leaders perform more emotional labor translate into an unspoken requirement that they *should do so*: In order to be seen as effective leaders, women (but not men) must be interpersonally sensitive—sympathetic, compassionate, understanding, forgiving, helpful. These demands to wield power “with a velvet glove” become sharply apparent in the backlash (i.e., social and economic penalties; Rudman, 1998) that high-power women encounter when they do not accommodate or spare others’ feelings. A plethora of studies following role congruity theory (Eagly and Karau, 2002) have demonstrated that female leaders are evaluated negatively when they enact their role in dominant ways—for instance, when they discipline or give negative feedback to subordinates (Sinclair and Kunda, 2000; Atwater et al., 2001; Brett et al., 2005) or when they demand a behavior change in others (Williams and Tiedens, 2016). These dominant behaviors, which are antithetical to emotional labor, lead to a perceived “communality deficit” in female leaders (Heilman and Okimoto, 2007; see also Ma et al., 2022), causing them to be seen as cold and interpersonally hostile (Heilman and Okimoto, 2007). Women (but not men) who lead with a directive style are more likely to receive negative evaluations than women who lead with a democratic style (Eagly et al., 1992), and abusive leadership tends to be penalized more harshly in female than in male leaders (Kim et al., 2021). When emotional labor is not readily apparent in leaders, those leaders fare worse if they are women.

The demands placed on female leaders focus strongly on the intrapersonal emotional labor aspect of *deamplifying* emotion—taming the expression of intense emotions (Matsumoto et al., 2005; Moran et al., 2013). Although research indicates that women and men report feeling most emotions to the same degree (Barrett et al., 1998; Else-Quest et al., 2012), women are stereotyped as *too emotional* and overly sensitive (Fischer, 1993; Shields, 2013; Dolan, 2014), and therefore unable to keep a cool head to make decisions rationally (e.g., Citrin and Roberts, 2004; see Brescoll, 2016, for a review). Thus, the emotional makeup of women is viewed as incompatible with some of the intrapersonal emotional labor requirements of high-level positions (Fischbach et al., 2015), leading to close scrutiny of female powerholders’ emotional expression. For example, women in top positions elicit more negative evaluations than men in similar roles for expressing anger (Lewis, 2000; Timmers et al., 2003; Brescoll and Uhlmann, 2008), a highly dominant emotion that is typically off limits for low-power individuals (Plant et al., 2000; Tiedens et al., 2000; Petkanopoulou et al., 2019) as well as powerful women (but tends to be condoned in high-power men). But the demand on powerful women to deamplify emotion for the benefit of others does not only target negative emotions, but all emotions more generally (for reviews, see Brescoll, 2016; Smith et al., 2016). As a result, women in high places walk a fine line, risking backlash from others if they fail to get emotional labor “just right.”

Research further suggests that performing emotional labor may allow women to successfully ascend the organizational hierarchy, eschewing the negative reactions they often encounter when they behave in explicitly dominant ways (Williams and Tiedens, 2016). For instance, some studies indicate that women reap more benefits than men from enacting transformational leadership practices, such that the teams they lead perform better (Chen and Shao, 2022). Men are held to a lower standard in this regard, as evidenced by research showing that men who practice transformational leadership tend to be evaluated as more promotable than women (Hentschel et al., 2018). These findings highlight the persistence of an impression management conundrum for women in top positions (Phelan and Rudman, 2010), and suggest the possibility that emotional labor practices may help women navigate these hurdles. Indeed, to lead and influence others without seeming overly domineering, women in powerful roles usually tame the way they express their power (e.g., Amanatullah and Morris, 2010; Moss-Racusin and Rudman, 2010; Brescoll, 2011; Amanatullah and Tinsley, 2013). Practicing emotional labor as an impression management strategy may result in a more prosocial use of power overall.

## The Downstream Consequences of Women’s Prosocial Use of Power

Women’s higher likelihood than men of engaging in prosocial behaviors when they occupy high-power roles has important consequences on many levels: for individual women, for subordinates who report to female authorities, and for organizations and society as a whole. The *positive* effects of women’s more prosocial use of power tend to benefit other people: Those who report to or work directly for them, as well as the organizations or groups in which women’s power is embedded. Importantly, a focus on the emotional labor practices that underlie prosocial behavior sheds light on the potential *negative* effects of women’s prosocial use of power, which burden individual women. We discuss these positive and negative downstream consequences first; then, we articulate how powerful women’s emotional labor practices may contribute to gender inequality in organizational hierarchies.

### The Positive Effects of Powerful Women’s Prosocial Behavior

Women’s more prosocial use of power is likely to confer many benefits for subordinates and organizations (Figure 1, *path j*). When those in powerful positions are interpersonally sensitive, subordinates directly reap the benefits—for example by being able to influence the decision-making process (De Wit et al., 2017). Organizations as a whole may benefit as well, as emotional labor is central to some of the key aspects of transformational leadership, like individualized consideration (Bass et al., 2003; Byrne et al., 2014), and it could foster a more socially responsible use of power (e.g., Chen et al., 2001). Similarly, in the context of political power and leadership, research has found that politicians’ tendency to practice emotional labor (for example, by employing courteous speech and avoiding incivility in debates) can be highly beneficial, reducing political polarization and increasing trust in politicians (Skytte, 2021).

For these reasons, women’s higher tendency than men to behave prosocially when they occupy high-power roles may confer important advantages on the people they lead and the organizations in which their power is embedded. Indeed, gender differences in leadership effectiveness tend to favor women over men (Eagly and Carli, 2003; Eagly et al., 2014; Offermann and Foley, 2020). Employee well-being tends to be higher in companies with more women in top positions (Thomas et al., 2021), and teams led by women tend to report more cohesion and cooperation (Post, 2015). Other research suggests that firms with more women in high-power roles are less likely to face discrimination lawsuits (Abebe and Dadanlar, 2021), have better financial performance (Glass and Cook, 2018; Hoobler et al., 2018), and engage in more socially responsible practices (e.g., Glass et al., 2016). In the political realm, a higher proportion of women in parliaments is associated with lower levels of corruption at the country level (Dollar et al., 2001; Swamy et al., 2001; Rivas, 2013). Although some of these findings may rely on observational data, raising the possibility of reverse causality, they converge with experimental studies that suggest a causal relationship. To illustrate, in a series of experiments, the mere presence of a female leader relative to a male leader caused people to anticipate fairer treatment within an organization and better personal outcomes because they associated stronger communal values in the organization when women occupied leadership roles (Joshi and Diekman, 2021). Similarly, when a hypothetical organization was in crisis, participants in two experiments were more likely to trust the organization (e.g., be willing to invest in it) when it was led by women than by men because they expected women to be more skilled at interpersonal emotion management (Post et al., 2019).

We argue that these advantages and benefits may stem from female leaders’ greater tendency to use their power in prosocial ways, and that male leaders (and the organizations that they lead) would also generally benefit from practicing more emotional labor. Regardless of their gender, powerholders who practice emotional labor can foster an environment in which employees and subordinates feel supported, are happier, and perform better (Thomas et al., 2021). Thus, although our focus in this article has been on the high standard for emotional labor against which female leaders are judged compared to male leaders (which is arguably unfair), perhaps a greater focus should be placed on identifying ways of increasing emotional labor among male leaders. Indeed, recent research indicates that both male and female leaders can enhance their effectiveness by enacting more communal behaviors that foster cooperation and trust (e.g., Gartzia and van Knippenberg, 2016; Hentschel et al., 2018; Gartzia and Baniandrés, 2019; Post et al., 2019). As more women attain high-power roles, their tendency to practice emotional labor might promote a shift in people’s implicit notions of leadership toward valuing communality as a central rather than peripheral aspect (Vial and Napier, 2018), one equally expected and rewarded in all leaders regardless of their gender.

### The Negative Effects of Powerful Women’s Prosocial Behavior

Whereas powerful women’s emotional labor tends to benefit other people, we propose that practicing emotional labor also entails costs for individual powerholders (Figure 1, *path k*). Although women may be socialized to practice emotional labor more than men (Brody, 1993; Hibbard and Buhrmester, 1998; Shields, 2002; Chaplin et al., 2005) and may come to develop stronger emotional abilities than men (e.g., Ickes et al., 2000; Cabello et al., 2016), they may still experience the added external demand to practice emotional labor as a burden. If women in power are interpersonally sensitive because they genuinely care for others (i.e., due to an internal motivation to behave in prosocial ways), then they might feel authentic and experience a higher sense of well-being than when they lack power (Kifer et al., 2013), due to an enhanced felt ability to fulfill their communal goals (Keltner et al., 2003; Guinote, 2007, 2008; Diekman et al., 2011). However, to the extent that women in power feel *pressured* to perform emotional labor (i.e., when they do so to avoid penalties for behaving too dominantly; Phelan and Rudman, 2010), emotional labor may take a psychological toll and detract from their well-being, making the exercise of power exhausting and emotionally draining for women.

Caring for other people can be burdensome in general. Other-oriented emotions such as empathy and compassion (i.e., the emotions that underlie prosocial behavior; Batson, 2011) are cognitively costly and effortful, and people tend to avoid feeling these emotions when given the chance (Cameron and Payne, 2011; Cameron et al., 2019; Scheffer et al., 2021). More specifically, research has documented how emotional labor can be psychologically costly for those who practice it: The purposeful self-control and the suppression of felt emotions that are often involved in the more intrapersonal aspects of emotional labor (Grandey, 2000) have been linked with intensified negative feelings (Scott and Barnes, 2011); emotional dissonance (Hopp et al., 2010); a reduced sense of authenticity (Brotheridge and Grandey, 2002); impaired memory (Richards and Gross, 2000); diminished task performance (Hülsheger and Schewe, 2011); reduced job satisfaction (Judge et al., 2009; Cheung and Tang, 2010); worsened mental health stemming from emotional exhaustion, stress, and burnout (Grandey, 2000; Brotheridge and Grandey, 2002; Beal et al., 2006; Johnson and Spector, 2007); and physical illness, including high blood pressure and cancer (Grandey, 2000; Johnson and Spector, 2007; Hopp et al., 2010).

As this litany suggests, if female powerholders perform more emotional labor than their male counterparts, then they may also fail to realize some of the benefits that power is supposed to bestow on well-being (Kifer et al., 2013). Indeed, women leaders are more likely to be exhausted and chronically stressed than men in similar positions (Thomas et al., 2021). A recent study further revealed that moving up in organizational rank was associated with greater emotional benefits for men than for women—i.e., diminished negative feelings of frustration and discouragement (Taylor et al., 2021). Other research indicates that, compared to men, women anticipate a higher burden of responsibility from attaining a high-power position as well as other negative outcomes (e.g., stronger stress and anxiety; Gino et al., 2015). It is possible that these negative effects may be countered by a sense of fulfillment or personal reward when emotional labor is internally motivated; however, if strong expectations for powerful women to be prosocial create an added pressure for them to engage in emotional labor, the evidence suggests that women will pay a psychological and physical toll.

## Implications for Gender Equality at the Top of the Hierarchy

In addition to the potential negative costs for individual women that we discussed in the previous section, emotional labor practices can create an uneven playing field that can contribute to gender inequality in organizational hierarchies (Figure 1, *path l*). Women continue to be greatly underrepresented in high-power roles (Eagly and Heilman, 2016; United Nations Women, 2021). Part of this underrepresentation is due to prejudice against women who deviate from the traditional gender role (Heilman and Eagly, 2008). For example, as mentioned earlier, there is strong evidence that women in roles of authority face more careful scrutiny than their male counterparts (Brescoll and Uhlmann, 2008; Barrett and Bliss-Moreau, 2009). But in addition to this prejudice, we argue that women’s stronger internal motivation to practice emotional labor, as well as the stronger external demands to do so that they experience relative to men, may undermine their likelihood of actually attaining and retaining power—helping maintain the unequal distribution of men and women in leadership roles.

### Channeling Women Toward Less Prestigious Leader Roles

The internal motivation to do emotional labor and enact power more prosocially may keep women from attaining the most prestigious high-level positions. Indeed, women appear more interested in high-power roles when the communal aspects of leadership are emphasized (Schneider et al., 2016; Pate and Fox, 2018; Schneider and Bos, 2019), which makes leadership and femininity appear more congruous (see also Henningsen et al., 2021; and Hentschel et al., 2021). However, such communal attributes are viewed as compatible with leadership primarily in “female-typed” domains such as healthcare or education rather than “male-typed” domains such as technology or finance (Cowgill and Vial, 2022), which tend to be viewed as much more prestigious and to be valued more (Block et al., 2018). Moreover, emotional labor practices may hinder women’s advancement up the management ladder, getting them stuck in mid-levels (e.g., International Labour Office, Bureau for Employers’ Activities, 2019; Einarsdottir et al., 2018). Emotional labor and prosocial work take up leaders’ finite time and energy resources, but are often “invisible” and not usually rewarded in formal ways in organizational contexts (Steinberg, 1999; Guy and Newman, 2004; Bolino and Grant, 2016). Relational service work in academia (e.g., mentoring or doing special favors for students), which female faculty tend to perform at higher rates than male faculty (Tunguz, 2016; Guarino and Borden, 2017; Hanasono et al., 2018; Berheide et al., 2022), is a chief example of the draining effects of such (invisible) emotional labor: Such work takes limited time away from other activities (e.g., research) that are valued much more highly in promotion and tenure decisions. Indeed, experiments show that women are more likely to volunteer for, be asked to perform, and accept requests to do “low-promotability” tasks that benefit organizations but are unlikely to impact career advancement into more senior leadership roles (Babcock et al., 2017). Thus, emotional labor practices may promote gender segregation *within* leadership, feeding a two-tier system in which male managers are at the top and female managers are at the bottom.

### Discouraging Women From Pursuing Top Roles

By making the psychological experience of power overall less appealing for women, the stronger emotional labor demands that women face could discourage them from pursuing high-power roles, ultimately maintaining male dominance in these roles. Again, the difference between internal and external motivation becomes crucial to understanding this nuanced distinction: Whereas women may be intrinsically drawn to high-power roles in contexts that favor communal behavior (Schneider et al., 2016; Pate and Fox, 2018; Schneider and Bos, 2019), they may nevertheless resent the strong external pressure to practice emotional labor around the clock. Research suggests that power is most appealing when it is construed in terms of personal opportunities, and less so when it is construed in terms of responsibility toward others (Sassenberg et al., 2014). If women, relative to men, construe power in a way that entails less autonomy and more responsibility for others, and if women in power experience stronger demands to toe a fine emotional line when dealing with others, these added burdens may partly explain why women are less interested than men in high-power roles. Women may see power as more of a “chore” than men—and rightly so, based on what can be gleaned from the literature on women’s experiences with high power roles (e.g., Brescoll, 2016; Thomas et al., 2021). Power, saddled by strict emotional labor demands, may not be an attractive prospect.

### Pushing Women to Opt Out of High-Power Roles

In addition to making power less appealing to women or guiding women toward less prestigious high-level roles, emotional labor can further undermine gender equality by making it difficult for women to retain their power. Even when they attain a high-power role, emotional labor makes exercising that power more exhausting and personally draining for women than for men, which may encourage them to give up or opt out of these roles. As reviewed earlier, emotional labor is costly both psychologically (Richards and Gross, 2000; Brotheridge and Grandey, 2002; Beal et al., 2006; Hopp et al., 2010; Scott and Barnes, 2011) and physically (Grandey, 2000; Johnson and Spector, 2007; Hopp et al., 2010), and has a negative impact on work-related outcomes such as performance and job satisfaction (Judge et al., 2009; Cheung and Tang, 2010; Hülsheger and Schewe, 2011). Qualitative studies have revealed that the difficult task of expressing one’s authentic self while acquiescing to external expectations to perform emotional labor may drive women to opt out of leadership positions (Frkal and Criscione-Naylor, 2020). Additional research is needed to provide quantitative data to support these findings and to further examine the mechanisms through which emotional labor demands push women out of high-power roles, promoting gender segregation at the top of organizations.

### Making Women’s Power More Precarious

Beyond the possibility that emotional labor demands may push women out of high-power roles, practicing emotional labor could make power more precarious, putting women at risk of losing it. One reason why women with power cannot simply ignore emotional labor demands from others is that the legitimacy of their power—the extent to which others feel that women deserve to be heeded as authorities—is usually in question (Vial et al., 2016). Even when they occupy a formal position in an organizational hierarchy that confers them with structural power and control over resources, it is more difficult for female authority figures than it is for men in the same positions to elicit status (i.e., respect, admiration, acceptance from others; Magee and Frasier, 2014). These status attributions are key to imbuing power with the kind of legitimacy that fosters cooperation from subordinates and followers (Tyler, 2002, 2006; Levi et al., 2009; Magee and Frasier, 2014). For female powerholders, foregoing emotional labor seems like a steadfast way to lose legitimacy in the eyes of others and, in turn, to be undermined and questioned (Butler and Geis, 1990; Koch, 2005).

Additionally, emotional labor demands may create a catch-22 for women. When they heed such demands, women may run the risk of having their power contested by being seen as inauthentic (Gardner et al., 2009) or too tentative (Forsyth et al., 1997; Bongiorno et al., 2014; Nandkeolyar et al., 2022). The strong communality implicitly conveyed by emotional labor may be seen as more suitable for the follower role than the leader role (Braun et al., 2017). Thus, women in high-power roles who engage in emotional labor may sometimes lose credibility as leaders, especially among male subordinates (Embry et al., 2008; Bongiorno et al., 2014) who have a stronger preference than female subordinates for dominant leaders (Koenig et al., 2011; Vial and Napier, 2018), and who overall appear less supportive of female leaders (e.g., Netchaeva et al., 2015; Vial et al., 2018). These inequalities in leader support contribute to gender segregation at the top of organizations.

Finally, expectations that women will practice emotional labor to a higher extent when they become leaders may result in them being appointed to highly precarious high-power roles—a phenomenon known as the “Glass Cliff” (Ryan and Haslam, 2005, 2007; Glass and Cook, 2016; Morgenroth et al., 2020). Emotional labor (e.g., responding to and managing others’ negative emotions) and a prosocial use of power (i.e., an interpersonally oriented leadership style) may be particularly relevant in crisis situations, making people more likely to support and promote women into high-level roles that are risky and uncertain (Ryan et al., 2011; Gartzia et al., 2012). These precarious appointments, however, can set female leaders up for failure—which may subsequently negatively impact the prospects of other aspiring women leaders. For example, Manzi and Heilman (2021) showed in a series of experiments that participants were less likely to appoint a female candidate to replace an unsuccessful female leader, whereas male candidates were judged independently from the previous leader’s performance. Thus, by encouraging the promotion of women into high-risk leadership positions, emotional labor demands and expectations may help maintain gender inequality in executive roles.

## Conclusion

Research indicates that women wield power in more prosocial, interpersonally sensitive ways than men. We propose that a focus on emotional labor can illuminate why men and women express power differently. The current review highlights multiple routes through which emotional labor practices underlie this gender difference, focusing on women’s stronger ability, internal motivation, and external demands to practice emotional labor. By distinguishing among these different routes, we shed light on the disparate consequences of women’s more prosocial use of power. Although it has benefits for other people, it also represents an important burden for women themselves—especially when it is motivated by external demands and the prospect of backlash. The stronger emotional labor demands placed on high-power women relative to high-power men can create an uneven playing field, helping explain why women continue to be sorely underrepresented in high-power roles.

## Author’s Note

We are grateful to Jenny Veldman at New York University Abu Dhabi for helpful comments on previous drafts of this manuscript.

## Author Contributions

AV conceptualized and wrote the first draft of the manuscript. CC contributed to the literature review and manuscript revision. Both authors approved the submitted version.

## Conflict of Interest

The authors declare that the research was conducted in the absence of any commercial or financial relationships that could be construed as a potential conflict of interest.

## Publisher’s Note

All claims expressed in this article are solely those of the authors and do not necessarily represent those of their affiliated organizations, or those of the publisher, the editors and the reviewers. Any product that may be evaluated in this article, or claim that may be made by its manufacturer, is not guaranteed or endorsed by the publisher.

## Footnotes

1 Throughout this article, we use the term *gender* to refer to the behavioral, social, and psychological characteristics of women and men (as well as norms and expectations about them), which are frequently associated with the biological categories of female and male (e.g., Deaux, 1985; Pryzgoda and Chrisler, 2000). We favor the term *gender* over the term *sex* in our writing because, although it is impossible to fully know the extent of biological and environmental influences on human behavior, the term *gender* has more inclusive implications than the term *sex* (Frieze and Chrisler, 2011).

2 Emotional labor practices can be combined with the unique behavioral affordances of high-power roles, manifesting in leadership styles that are more interpersonally oriented, as we explain in the section on *Emotional Labor at the Top of the Hierarchy: Women’s Prosocial Use of Power.*
